# Can Not Be Sent by Mail

**Published:** 1895-04

**Authors:** 


### Can Not be Sent by Mail.

Bacteriologists and pathologists should take notice that by the new postal regulations, "disease germs and matters from diseased persons" are unmailable matter, and can not in the United States be sent by post.

